# Flow Matters: Distal Limb Perfusion in Extracorporeal Membrane Oxygenation

**DOI:** 10.1016/j.jaccas.2026.107540

**Published:** 2026-03-31

**Authors:** Mark S. Slaughter, Gretel Monreal, Joshua G. Crane

**Affiliations:** Department of Cardiovascular and Thoracic Surgery, University of Louisville, Louisville, Kentucky, USA

**Keywords:** perfusion, peripheral circulation, peripheral vascular disease

**Figure:**
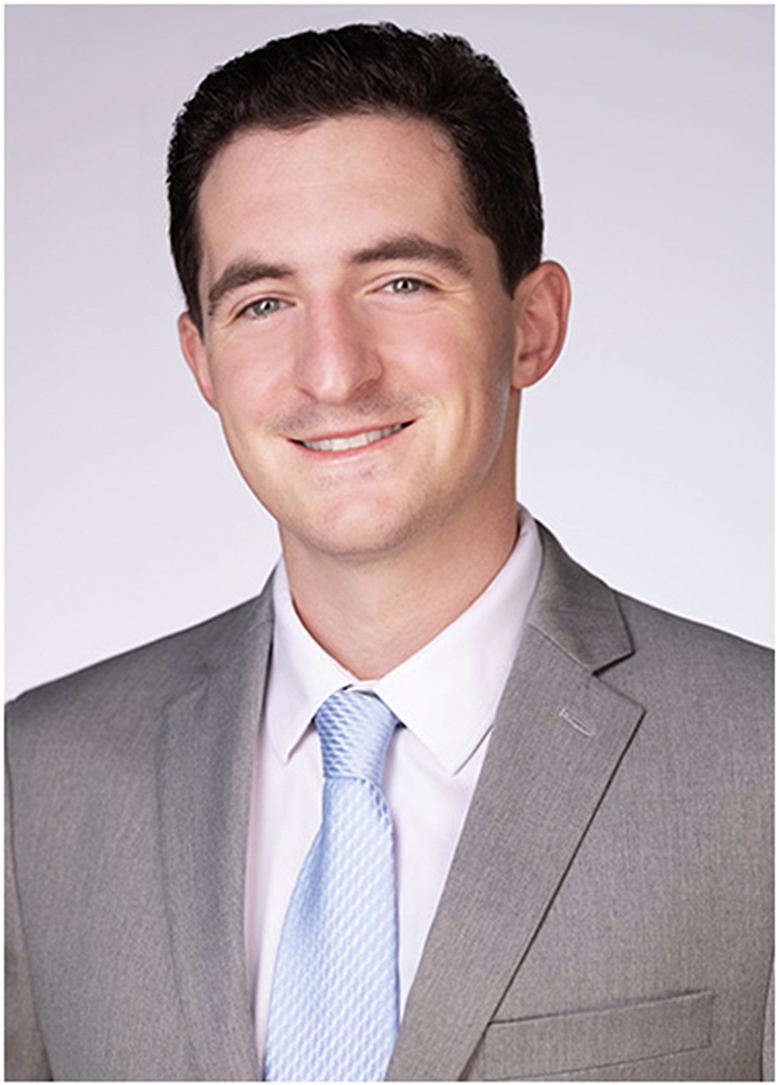

Limb ischemia is the most common vascular complication in patients receiving femoral venoarterial extracorporeal membrane oxygenation (VA-ECMO) and is associated with an increased risk of mortality.^1^^,^^2^ To improve blood flow distal to the arterial cannula, the Extracorporeal Life Support Organization recommends placement of a wire-reinforced introducer sheath as a distal perfusion catheter (DPC); however, no consensus exists regarding the timing, type, or size of the DPC.^3^, ^4^, ^5^ In their recent article, Fadel et al^6^ describe novel use of the Catapult Guide Sheath (Boston Scientific), which has a detachable hemostatic valve/sidearm, as a DPC for use in patients receiving VA-ECMO.

The authors report flow measurements with and without removal of the detachable hemostatic valve/sidearm in 5 patients receiving femoral VA-ECMO who underwent 6-F Catapult placement as a DPC and found that removal of the hemostatic valve/sidearm enabled 220% greater flow. This article demonstrates successful use of the Catapult as a DPC to support distal limb flow in femoral VA-ECMO.

Traditional introducer sheaths are designed for percutaneous delivery of intravascular catheters and tools (via the hemostatic valve) and for pressure monitoring, blood sampling, and administration of various agents through the sidearm. They are not necessarily designed to deliver blood flow to support distal limb perfusion in VA-ECMO. Using a dynamic mock flow loop model of adult femoral VA-ECMO, we previously investigated the hydrodynamics and hemodynamics of use of pediatric arterial cannulas vs introducer sheaths as a DPC in femoral VA-ECMO and found that: 1) using a cannula specifically designed for delivering blood flow, such as a pediatric arterial cannula, results in more favorable distal limb hemodynamics over using an introducer sheath; and 2) the integrated stopcock on the sidearm of the introducer sheath and the small 90° sidearm attachment to the sheath hub are choke points restricting/limiting flow owing to their tiny holes, which negate the potential benefit of using a larger-sized sheath and which may be insufficient to provide distal limb perfusion as a DPC.^7^^,^^8^

We commend the authors on their efforts and success in identifying ways to improve distal limb flow in patients receiving VA-ECMO. Continued efforts should be made to identify best-practice standards for DPC strategies in femoral VA-ECMO.

## Funding Support and Author Disclosures

Drs Slaughter and Monreal have been investigators on National Institutes of Health (NIH) grant R44HL144214 unrelated to this report. Dr Slaughter has been an advisor to NorthStar Medical and is on the advisory board of Medtronic. Dr Monreal has received funds from the National Board of Medical Examiners (NBME) Medical Student Organization Assistance Initiative to support the Innovation in Medicine medical student interest group at the University of Louisville, has received honoraria from the European Science Foundation, and has been supported in part by a gift from Robert M. Prizant to the Legacy Foundation of Kentuckiana. Dr Crane has been supported by the Mansbach Research Endowment Fund at the University of Louisville.
